# Supplementary material for: Environmental drivers of the resistome across the Baltic Sea
Source: Microbiome. 2025 Apr 7;13:92. doi: 10.1186/s40168-025-02086-x (PMC11974054; doi:10.1186/s40168-025-02086-x)
Supplement: Supplementary file 2 — Additional file 1 (Supplementary-Information.pdf): Supplementary Fig. S1 Log-transformed values of the environmental factors visualized per region. Different letters indicate differences between groups tested by ANOVA (p-value <0.05). Supplementary Fig. S2 Comparison of ARG database and methods. Bar plots of A the number of annotations per method/database, B ADONIS R2 based on the relative influence of Region on the community dissimilarity between methods, and C the adjusted R2of the distance-based redundancy analyses (dbRDA). The *** in the adonis results indicate significance at p-value <0.001, and ** at p-value <0.01. Supplementary Fig. S3 Autocorrelations among the environmental factors. Supplementary Fig. S4 Relative abundance plots of A ARG types, B microbial phyla, C MRGs, D MGE, and E VFG categories. Supplementary Fig. S5 A Relative abundance boxplots of the top ARG types across regions. Separate boxplot visualizations of the multidrug and glycopeptide resistance types in Fig. 1D. B Differential abundance test by Kruskal-Wallis rank sum and random forest. The variable importance plot shows the top 15 differentially abundant ARG subtypes with high mean decrease Gini scores. Supplementary Fig. S6 Differential abundance test by Kruskal-Wallis rank sum and random forest. The variable importance plot shows the 47 differentially abundant species. Supplementary Fig. S7 Alpha diversity of the A–B microbial communities, C–E MRG, F–H MGE, and I–K VFG. Different letters indicate differences between groups tested by ANOVA or Kruskal-Wallis test (p-value < 0.05). Supplementary Fig. S8 A Relative abundance boxplots of the top MRG types across regions. Separate boxplot visualizations of the B arsenic resistance protein ArsB and C copper-translocating P-type ATPase. Different letters indicate differences between groups tested by ANOVA (P-value < 0.05). Supplementary Fig. S9 Distance decay relationship of the Bray-Curtis dissimilarities of the ARG, microbial community, MRG, [file 40168_2025_2086_MOESM1_ESM.docx]

# Supplementary Information for

**Environmental drivers of the resistome across the Baltic Sea**

### Authors & Affiliations

Joeselle M. Serrana^1,2^*, Francisco J. A. Nascimento^1,3^, Benoît Dessirier^1,4^, Elias Broman^1,3,4^, and Malte Posselt^1,2^

^1^Stockholm University Center for Circular and Sustainable Systems (SUCCeSS), Stockholm University, 106 91 Stockholm, Sweden

^2^Department of Environmental Science (ACES), Stockholm University, 106 91 Stockholm, Sweden

^3^Department of Ecology, Environment, and Plant Sciences (DEEP), Stockholm University, 106 91 Stockholm, Sweden

^4^Baltic Sea Centre, Stockholm University, Stockholm, Sweden

*Correspondence: [Joeselle.Serrana@aces.su.se](mailto:Joeselle.Serrana@aces.su.se)

### Supplementary Figures

#### Supplementary Fig. S1 Log-transformed values of the environmental factors visualized per region. Different letters indicate differences between groups tested by ANOVA (p-value <0.05).

#### Supplementary Fig. S2 Comparison of ARG database and methods. Bar plots of A the number of annotations per method/database, B ADONIS R^2^ based on the relative influence of Region on the community dissimilarity between methods, and C the adjusted R^2^ of the distance-based redundancy analyses (db-RDA). The *** in the adonis results indicate significance at p-value <0.001, and ** at p-value <0.01.

#### Supplementary Fig. S3 Autocorrelations among the environmental factors.

#### Supplementary Fig. S4 Relative abundance plots of A ARG types, B microbial phyla, C MRGs, D MGE, and E VFG categories.

#### Supplementary Fig. S4 (cont.) Relative abundance plots of A ARG types, B microbial phyla, C MRGs, D MGE, and E VFG categories.

#### Supplementary Fig. S5 A Relative abundance boxplots of the top ARG types across regions. Separate boxplot visualizations of the multidrug and glycopeptide resistance types in Fig. 1D. B Differential abundance test by Kruskal-Wallis rank sum and random forest. The variable importance plot shows the top 15 differentially abundant ARG subtypes with high mean decrease Gini scores.

#### Supplementary Fig. S6 Differential abundance test by Kruskal-Wallis rank sum and random forest. The variable importance plot shows the 47 differentially abundant species.

#### Supplementary Fig. S7 Alpha diversity of the A-B microbial communities and their distribution pattern by C Shannon diversity, and D Pielou evenness across the Baltic Sea regions in haversine distance (km). Alpha diversity of the E-G predicted MRG, H-I MGE, and K-M VFG. Different letters indicate differences between groups tested by ANOVA or Kruskal-Wallis test (p-value < 0.05).

#### Supplementary Fig. S7 (cont.) Alpha diversity of the A-B microbial communities and their distribution pattern by C Shannon diversity, and D Pielou evenness across the Baltic Sea regions in haversine distance (km). Alpha diversity of the E-G predicted MRG, H-I MGE, and K-M VFG. Different letters indicate differences between groups tested by ANOVA or Kruskal-Wallis test (p-value < 0.05).

#### Supplementary Fig. S8 A Relative abundance boxplots of the top MRG types across regions. Separate boxplot visualizations of the B arsenic resistance protein *ArsB* and C copper-translocating P-type ATPase. Different letters indicate differences between groups tested by ANOVA (P-value < 0.05) .

#### Supplementary Fig. S9 Distance decay relationship of the Bray-Curtis dissimilarities of the ARG, microbial community, MRG, MGE, and VFG profiles with haversine distance (in km) in the Baltic Sea without the dead zones samples.

#### Supplementary Fig. S10 Effects coefficients of the PLS-PM model of the microbial community, ARG, MRG, MGE, and VFG profiles as latent variables.

## References

Alcock, B. P., Raphenya, A. R., Lau, T. T., Tsang, K. K., Bouchard, M., Edalatmand, A., ... & McArthur, A. G. (2020). CARD 2020: antibiotic resistome surveillance with the comprehensive antibiotic resistance database. *Nucleic Acids Research*, 48(D1), D517-D525.

Arango-Argoty, G., Garner, E., Pruden, A., Heath, L. S., Vikesland, P., & Zhang, L. (2018). DeepARG: a deep learning approach for predicting antibiotic resistance genes from metagenomic data. *Microbiome*, 6, 1-15.

Bonin, N., Doster, E., Worley, H., Pinnell, L. J., Bravo, J. E., Ferm, P., ... & Boucher, C. (2023). MEGARes and AMR++, v3. 0: an updated comprehensive database of antimicrobial resistance determinants and an improved software pipeline for classification using high-throughput sequencing. *Nucleic Acids Research*, 51(D1), D744-D752.

Bortolaia, V., Kaas, R. S., Ruppe, E., Roberts, M. C., Schwarz, S., Cattoir, V., ... & Aarestrup, F. M. (2020). ResFinder 4.0 for predictions of phenotypes from genotypes. *Journal of Antimicrobial Chemotherapy*, 75(12), 3491-3500.

Buchfink, B., Reuter, K., & Drost, H. G. (2021). Sensitive protein alignments at tree-of-life scale using DIAMOND. *Nature Methods*, 18(4), 366-368.

Feldgarden, M., Brover, V., Gonzalez-Escalona, N., Frye, J. G., Haendiges, J., Haft, D. H., ... & Klimke, W. (2021). AMRFinderPlus and the Reference Gene Catalog facilitate examination of the genomic links among antimicrobial resistance, stress response, and virulence. *Scientific Reports*, 11(1), 12728.

Gschwind, R., Ugarcina Perovic, S., Weiss, M., Petitjean, M., Lao, J., Coelho, L. P., & Ruppé, E. (2023). ResFinderFG v2. 0: a database of antibiotic resistance genes obtained by functional metagenomics. *Nucleic Acids Research*, 51(W1), W493-W500.

Lakin, S. M., Kuhnle, A., Alipanahi, B., Noyes, N. R., Dean, C., Muggli, M., ... & Boucher, C. (2019). Hierarchical Hidden Markov models enable accurate and diverse detection of antimicrobial resistance sequences. *Communications Biology*, 2(1), 294.

Martiny, H. M., Pyrounakis, N., Petersen, T. N., Lukjančenko, O., Aarestrup, F. M., Clausen, P. T., & Munk, P. (2024). ARGprofiler—a pipeline for large-scale analysis of antimicrobial resistance genes and their flanking regions in metagenomic datasets. *Bioinformatics*, 40(3), btae086.

Pei, Y., Shum, M. H. H., Liao, Y., Leung, V. W., Gong, Y. N., Smith, D. K., ... & Lam, T. T. Y. (2024). ARGNet: using deep neural networks for robust identification and classification of antibiotic resistance genes from sequences. *Microbiome*, 12(1), 84.

Yin, X., Jiang, X. T., Chai, B., Li, L., Yang, Y., Cole, J. R., ... & Zhang, T. (2018). ARGs-OAP v2. 0 with an expanded SARG database and Hidden Markov Models for enhancement characterization and quantification of antibiotic resistance genes in environmental metagenomes. *Bioinformatics*, 34(13), 2263-2270.
